# Consensus statement on standardizing CGM evaluation metrics in Latin America: an expert approach

**DOI:** 10.1186/s13098-025-01851-0

**Published:** 2025-07-21

**Authors:** Mauro Scharf, Karen Feriz, Carlos Yepes, Álvaro Contreras, Lorena Lequi, David Sanhueza, Marcio Krakauer, Eduardo Márquez, Matías Ré, Roopa Mehta

**Affiliations:** 1 Pediatric Endocrinology Unit, Centro de Diabetes Curitiba, Hospital Nossa Senhora das Graças, Curitiba, Brazil; 2 Endocrinology Service, Fundación Valle del Lili University Hospital, Universidad ICESI, Cali, Colombia; 3 Department of Endocrinology, Hospital Universitario Clínica San Rafael and Hospital Universitario San Ignacio, Bogotá, Colombia; 4 Department of Diabetology, Clínica Universidad de los Andes, Santiago, Chile; 5 Mains Bleues Diabetes, Centro de Investigación diabetes, metabolismo y enfermedades asociadas, Rafaela, Santafe Argentina; 6 Department of Diabetology, Universidad Andrés Bello and Hospital y CRS El Pino, Santiago, Chile; 7 Diabetes Division, Liga de Diabetes, Faculty of Medicine, ABC Medical School, São Paulo, Brazil; 8 Department of Endocrinology, Instituto Jalisciense de Metabolismo, Guadalajara, Mexico; 9 Department of Diabetology, CINME (Centro de Investigaciones Metabólicas), Buenos Aires, Argentina; 10 Department of Endocrinology and Metabolism and UIEM, Instituto Nacional de Ciencias Médicas y Nutrición Salvador Zubirán, Mexico City, Mexico

**Keywords:** Consensus statement, Continuous glucose monitoring, CGM regulation, Latin America, Diabetes management, Accuracy standards, Clinical validation, Patient safety

## Abstract

**Background:**

Latin America has no accepted performance standards for continuous glucose monitoring (CGM) technology evaluation. This has resulted in the emergence of various CGM devices in the market that do not meet strict quality, accuracy, reliability or safety standards. CGM systems are crucial for managing diabetes, as they provide frequent glucose measurements and help detect hypoglycemia or hyperglycemia episodes or even predict these events. Ensuring the reliability and accuracy of CGM devices is essential for patient safety. This consensus statement aims to establish a consensus-driven framework of expert recommendations regarding the metrics that should be evaluated to achieve high standards in CGM devices.

**Materials and methods:**

A modified Delphi methodology was employed, engaging endocrinologists, pediatric endocrinologists and diabetologists from Latin America. Experts participated in multiple rounds of surveys and discussions to reach consensus on key characteristics measures, including accuracy thresholds, clinical validation protocols, and post-market surveillance requirements. Quantitative and qualitative data were analyzed to ensure robust recommendations.

**Results:**

The expert panel identified major gaps in existing CGM regulations and established 12 key recommendations and one checklist to align Latin American standards with international best practices. These included the implementation of minimum accuracy thresholds, the adoption of standardized clinical validation protocols, and the enforcement of post-market surveillance measures. The panel also emphasized the importance of patient education, healthcare provider involvement in decision-making, and accessibility to enhance CGM adoption and usability. We underscore the necessity of these measures to improve patient outcomes, patient safety, and regulatory consistency in the region, while also enhancing CGM reliability and accuracy.

**Conclusion:**

This consensus statement highlights the urgent need for a standardized metrics to evaluate CGM devices in Latin America. Implementing standardized accuracy requirements, rigorous validation protocols, and enhanced patient education will ensure device reliability, improve clinical outcomes, and foster a more equitable healthcare landscape for diabetes management in the region.

## Introduction

Continuous glucose monitoring (CGM) systems have revolutionized diabetes management by providing real-time glucose readings, allowing users to identify patterns, trends, and fluctuations throughout the day and night. By offering detailed insights into glucose dynamics, CGM systems support patients and healthcare providers in making more informed treatment decisions, ultimately improving glycemic control [1, 2]. Their widespread adoption is largely driven by the potential to mitigate acute complications associated with hyper- and hypoglycemia, ultimately improving patient longterm outcomes. In both type 1 and type 2 diabetes, CGM has helped improve overall glycemic control, which can lead to better HbA1c levels [3, 4]. The ability to continuously monitor glucose levels reduces the burden of frequent fingerstick testing and allows for timely therapeutic adjustments, further enhancing diabetes self-management and quality of life.

Unlike in the United States—where most CGM sensors are regulated as Class III devices that require Pre-Market Approval (PMA), and only those that meet the FDA’s Integrated CGM (iCGM) special-control criteria may use the lower-risk 510(k) pathway (e.g., FreeStyle Libre 2 Plus is an iCGM, whereas Medtronic Guardian Sensor 3 remains a non-iCGM PMA device)— [5], Latin American regulatory frameworks, and Europe as well (where an eCGM pathway is still under development), have not yet established standardized performance evaluation criteria tailored to CGM technology [6]. This regulatory gap raises concerns about the performance security, accuracy and clinical reliability of devices available in the region.

Although CGM technology has demonstrated substantial clinical benefits, significant gaps remain in ensuring standardization, reliability, and validation across diverse patient populations and clinical settings [5, 7]. Current regulatory pathways in Latin America do not consider standardized accuracy thresholds evaluation, robust clinical data, or transparent reporting of real-world performance data [6, 8–10]. Existing studies evaluating CGM performance often employ heterogeneous design and methodologies, different glucose values and limited sample populations, making direct comparisons between different systems challenging [6, 11]. Furthermore, while the FDA’s iCGM special control performance requirements establish specific accuracy and precision benchmarks, there is no globally accepted performance standard for CGM, leading to potential inconsistencies in device performance [8, 10]. Thus, there is a pressing need for a standardized approach to evaluating CGM device performance and ensuring safety across diverse user groups in Latin America.

This consensus statement advocates for the establishment of a structured framework that defines minimum performance requirements for CGM devices, aligning Latin American CGM standards with FDA’s iCGM special-control benchmarks to ensure accurate, user-friendly, and interoperable devices that foster patient adherence and guarantee safety [5, 11]. We assert that standardized evaluation criteria are essential to improving device reliability, facilitating cross-device comparisons, and enhancing patient safety. The pivotal questions guiding this consensus statement are: [1] What minimum accuracy and precision thresholds should be assigned for CGM devices approval in Latin America? [2] How can clinical validation protocols be optimized to reflect real-world usage patterns? [3] What evaluation criteria measures can bridge the gap between Latin America and global CGM device standards? Addressing these questions will contribute to a more robust and harmonized regulatory framework for CGM technology in the region.

To support this consensus statement, we leveraged a structured expert consensus methodology using the modified Delphi approach, engaging Latin American endocrinologists, pediatric endocrinologists and diabetologists. This process ensured the inclusion of diverse perspectives to develop a pragmatic and comprehensive framework for CGM standardized evaluation. The expert consensus approach strengthens the validity of our position by integrating real-world data with clinical expertise. By advocating for clear regulatory benchmarks, this consensus statement aims to inform policymakers, manufacturers, and healthcare professionals about the urgent unmet need for a minimum set of requirements for clinical testing and performance metrics for CGM systems [12], ultimately fostering a more reliable and patient-centered approach to diabetes technology regulation in Latin America.

## Materials and methods

### Study design

This consensus statement employed a modified Delphi method to establish a consensus-driven framework for CGM device regulation. A panel of Latin American experts, including endocrinologists, pediatric endocrinologists and diabetologists, convened to establish an agreement on key clinical regulatory evaluation metrics. Experts were selected based on their clinical and research expertise in CGM technology and diabetes management. The inclusion criteria required a minimum of five years of experience in relevant fields and active participation in diabetes research or policy development, Experts were purposively selected as key opinion leaders (KOLs) in CGM and diabetes care, on the basis of their recognized clinical leadership, frequent participation as faculty in regional congresses, and authorship of peer-reviewed publications or clinical guidelines. Each candidate was identified by the steering committee through professional networks and then invited individually.

**Fig. 1:**
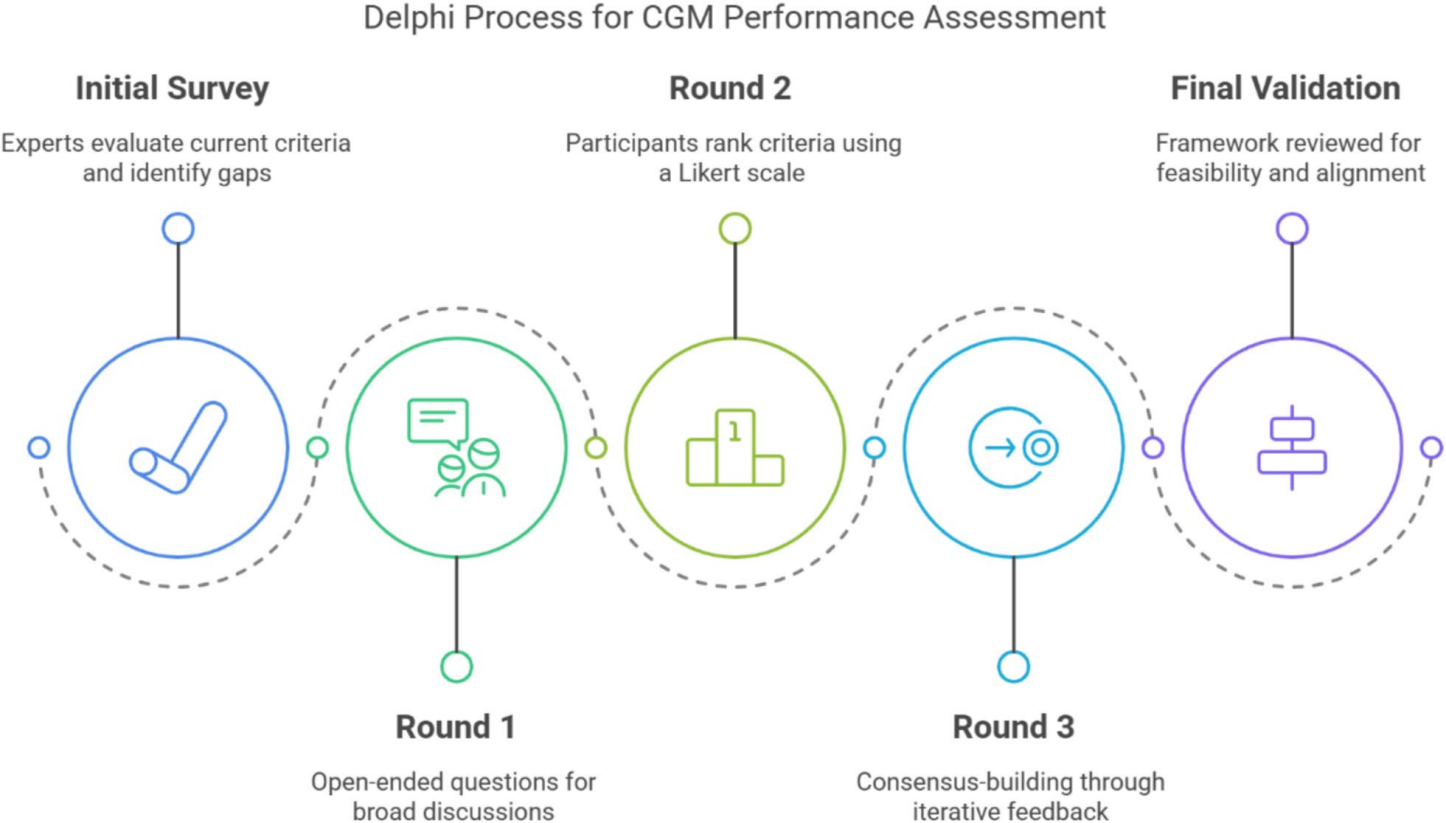
Delphi Process for CGM Performance Assessment. This figure was created by the authors to illustrate the Delphi methodology used in the CGM performance assessment. It was not adapted from any published source

The Delphi process consisted of multiple questionnaires rounds to refine and validate evaluation criteria Fig. 1

### Data collection and analysis

Quantitative and qualitative data were collected at each Delphi round. Thematic analysis was used to synthesize expert opinions, while statistical methods (e.g., median ranking and interquartile range calculations) assessed consensus levels. A predefined agreement threshold of 75% was set to determine consensus [13].

### Ethical considerations

All participants provided informed consent, and the consensus process adhered to ethical guidelines for expert-based research. To mitigate potential biases, the panel included diverse stakeholders from various clinical backgrounds across Latin America. Their responses were anonymized to ensure confidentiality.

This structured methodology ensures that the proposed regulatory framework for CGM devices is evidence-based, feasible, and aligned with international best practices while addressing the unique challenges of the Latin American regulatory environment.

## Results

### Expert consensus panel

The expert consensus panel included ten specialists from five Latin American countries, representing a diverse range of expertise in diabetology and endocrinology. The panelists were from Argentina (La Plata and Rafaela), Chile (Santiago de Chile), Colombia (Cali, Bogotá), México (Ciudad de México, Guadalajara), and Brazil (São Paulo, Curitiba). Their backgrounds encompass adult and pediatric endocrinology, as well as diabetology with extensive clinical experience in CGM technologies.

### Recommendations

**Fig. 2:**
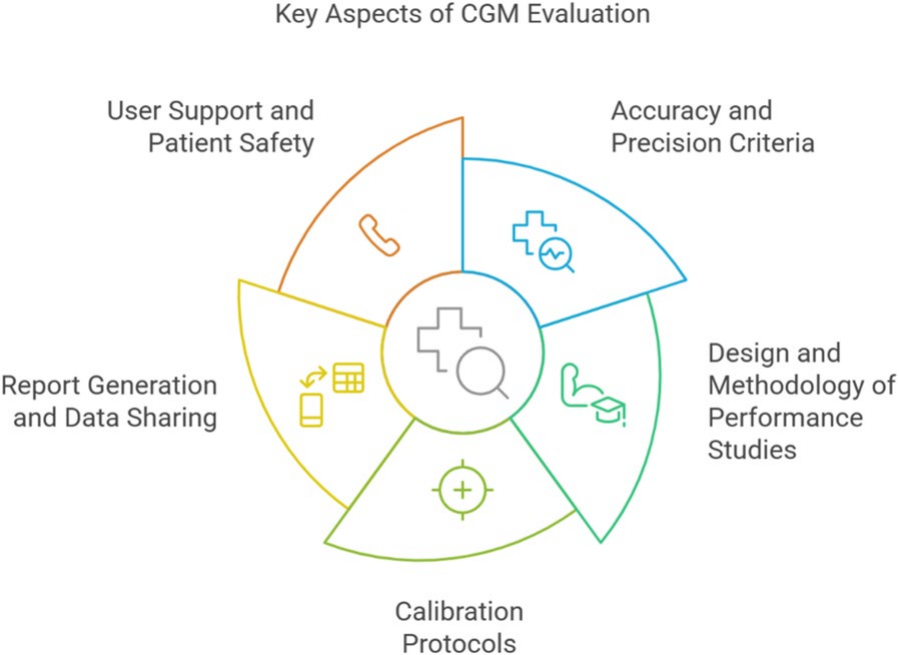
Key Aspects of CGM Evaluation. This figure was created by the authors to illustrate the main factors considered in CGM evaluation. It was not adapted from any published source

Through a structured Delphi process, the panelists systematically assessed key aspects of CGM evaluation, evaluation, achieving unanimous consensus (100% agreement) across all five critical topics Fig. 2:

#### Accuracy and precision criteria

**Table:** 

Key Points • CGM devices must adhere to strict accuracy standards for clinical reliability • A multidimensional evaluation of accuracy must jointly consider MARD, Consensus Error Grid, and Agreement Rate to ensure clinical relevance • Compliance with iCGM Special-Control Performance Requirements contributes to precise and reliable glucose measurements by establishing minimum accuracy benchmarks • Accuracy must be maintained throughout the sensor’s lifespan to ensure sustained performance • Validation across the full glycemic range, including hypo- and hyperglycemia, is critical • Device accuracy and safety must be evaluated in diverse patient populations • Implementation in Latin America would require a multi-step process involving pre-submission dialogue, agreement on study protocols, and post-market surveillance coordinated by national regulators together with a regional expert working group	

### Recommendations

**Fig. 3:**
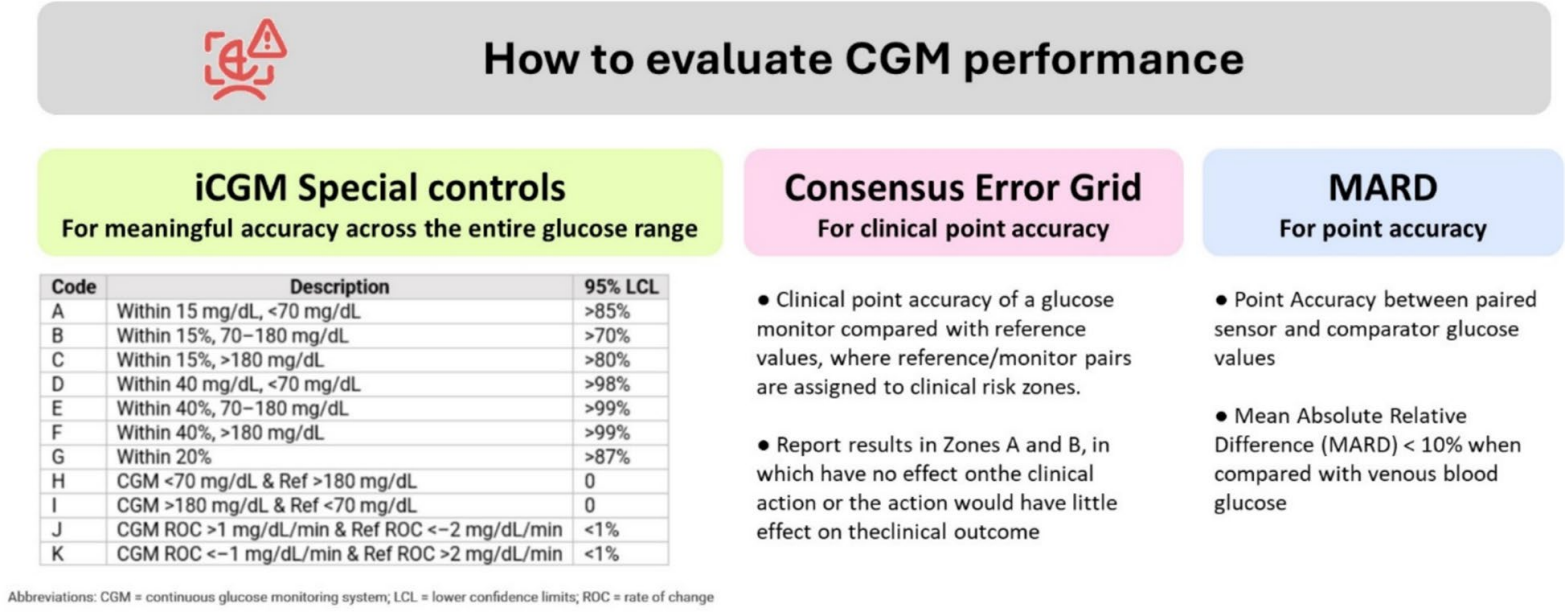
CGM Performance Evaluation: Accuracy and Precision Criteria. This figure was developed by the authors and includes agreement rate data reproduced from a published source for illustrative purposes [5]. All other components of the figure are original

1. Manufacturers of CGM devices should demonstrate accuracy by reporting standardized metrics, including: Mean Absolute Relative Difference (MARD); the consensus error grid; and the agreement rate stablished by iCGM Special Control (Fig. 3).
2. Practitioners should prioritize using iCGM Special Control Performance Requirements systems when selecting CGM for patient care.

### Rationale

iCGM Special Control Performance Requirements enhance patient safety and interoperability with insulin pumps, smart insulin pens, and digital health platforms, allowing for automated insulin delivery and more effective glycemic control [5, 14]. Yet integration alone should not determine CGM choice; sustained accuracy and reliability, strict accuracy standards are essential for the classification of CGM systems, requiring are paramount. Harmonized benchmarks such as Mean Absolute Relative Difference (MARD) to assess deviations from reference values, the percentage of readings within 15/15%, 20/20%, and 40/40% zones, trend accuracy and Consensus Error Grid analysis to evaluate clinical reliability [15]. In accordance with the IFCC Working Group on CGM, performance studies must also report accuracy stratified by rates of glucose change (for example ≤ 1, 1–2, > 2 mg dL⁻^1^ min⁻^1^) to verify sensor behavior during rapid glycaemic excursions [16]. Acceptance thresholds should therefore be defined together with a standardized study protocol that guarantees adequate sampling across both glucose ranges and rate-of-change strata [16]. Regulatory frameworks should focus on defining appropriate performance criteria that all CGM systems must meet to ensure their clinical effectiveness and safety.

To maintain high clinical reliability, CGM devices must adhere to clearly defined quality standards and minimum performance thresholds [1, 17, 18]. Current regulatory landscapes lack uniformity in specifying accuracy and precision requirements, leading to variability in device performance across different markets [8]. Establishing and enforcing standardized performance criteria can ensure that all CGM systems meet a baseline level of safety and efficacy [7, 19]. Additionally, manufacturers should be required to conduct rigorous pre-market and post-market performance evaluations to verify compliance with these standards [12].

Accuracy over the full lifespan of a CGM sensor is crucial for maintaining trust in the technology system and ensuring effective diabetes management [20]. The MARD is a widely used metric for CGM accuracy, yet its reporting often lacks consistency across studies and regulatory submissions [20, 21]. Specifically, MARD values should always be reported in the context of the population being studied, number of paired measurements and the glycemia comparator used, in addition to other factors that may impact the value of the MARD [22]. A CGM system with an initially low MARD but significant accuracy degradation over time may fail to provide reliable glucose readings, particularly in the latter days of sensor wear [20]. To address this, performance evaluations should require MARD reporting from the first to the last day of use, capturing any decline in accuracy [21, 23]. This would ensure that regulatory approvals and clinical recommendations are based on a more comprehensive assessment of device performance. These measures would help healthcare providers make informed choices about which CGM systems to prescribe and guide patients toward the most dependable options.

Beyond sensor stability, CGM accuracy must be validated across the full spectrum of glucose levels to ensure real-world applicability [24–26]. To fully reflect the user experience, validation studies must also collect comparator measurements during both stable periods and rapid transitions. In line with the IFCC Working Group on CGM [16], at least 7.5% of all values should cover each of the following situations:

(a) BG < 70 mg/dL;

(b) BG > 300 mg/dL;

(c) BG ≥ 70 mg/dL with rate of change < –1 mg/dL/min or BG < 70 mg/dL within 30 minutes at the current rate of change; and

(d) BG ≤ 300 mg/dL with rate of change > +1.5 mg/dL/min or BG > 250 mg/dL within 30 minutes at the current rate of change.

Many CGM performance studies primarily report accuracy within a narrow glucose range, often not including extreme hypo- and hyperglycemic conditions where accurate readings are most critical [14, 25, 27]. In practice, patients with diabetes experience substantial glycemic variability, making it essential that CGM systems perform reliably across all physiological glucose concentrations. Regulatory requirements should mandate that manufacturers report accuracy data across predefined glucose ranges, including Level 1 hypoglycemia is defined as a blood glucose concentration below 70 mg/dL (< 3.9 mmol/L) but at least 54 mg/dL (≥ 3.0 mmol/L), level 2 hypoglycemia occurs when blood glucose drops below 54 mg/dL (< 3.0 mmol/L) and level 3 hypoglycemia is the most severe form, characterized by altered mental and/or physical functioning that requires assistance from another person for recovery, regardless of glucose level [28]. When evaluating CGM systems for specific populations (e.g. pediatric patients, pregnant individuals, renal insufficiency), accuracy, user acceptability, and safety must be evaluated and data available for these groups [2]. Ensuring comprehensive performance validation would strengthen confidence in CGM systems and enhance their clinical utility across a wide range of patient scenarios.

#### Design and methodology of performance studies

**Table:** 

Key points • CGM studies require ethical approval and standardized study design—Clear inclusion/exclusion criteria, sample size justification, and study site descriptions are essential • Study design should ensure clinical applicability through rigorous methodological approaches, including robust statistical analysis and transparent reporting in scientific journals • Adequate sample size ensures statistical validity—Performance assessment studies typically include around 100 participants, generating approximately 12,000 paired glucose readings to ensure sufficient statistical power. For pediatric studies, 4,000 paired readings are a reasonable estimate, though data collection constraints may limit the ability to meet adult performance requirements • Testing should assess performance across a full glycemic range—Accuracy validation must cover hypoglycemia, euglycemia, and hyperglycemia under controlled conditions • Validation under real-world conditions is essential—CGM systems must be tested under exercise, meals, medication use, and other physiological variations to reflect real-world applicability	

### Recommendations

3. CGM performance studies should include:
A. **General Study Design**
  Ethical approval for the study.
  Clear inclusion and exclusion criteria, including potential interfering medications.
  Sample size justification.
  Description of investigational sites (single-center or multicenter).
  Role of the funding source.
B. **Study Population**
  Ensure the population reflects the intended use population regarding HbA1c, BMI, age, sex, ethnicity, type of diabetes mellitus, duration, and treatment.
  Population limits for CGM studies
    In CGM approval studies for type 1 diabetes, the proportion of participants with type 2 diabetes using insulin should not exceed ≤ 2% for those under 18 years old and ≤ 30% for those aged 18 years or older [14]
  Use a representative sample for real-world applicability.
C. **CGM System Use**
  Specify the number of CGM systems and sensors:
    Number of distinct CGM systems evaluated
    Number of sensors used (simultaneously and consecutively).
    Sensor manufacturing lots used.
  Report planned sensor wear days and intended sensor lifetime.
  Describe the protocol for sensor insertion, including the anatomical site and procedures for replacing failed sensors.
  Define calibration protocols, including the source and concentration of calibrators, if the CGM is not factory calibrated.
D. **Comparator Measurement Approach**
  Sampling compartment (capillary, venous, or arterialized-venous).
  Provide details on any relevant sampling protocols, including arterialization if applicable for the study context, while ensuring accurate glucose measurement.
  Glucose measurement methods and devices for comparator data.
  Harmonization procedures for comparator measurements (if applicable).
  Compliance with analytical performance specifications [14]
E. **Testing Procedures**
  Study setting: in-clinic, free-living, or hybrid.
  Report in-clinic session details, including:
    Number and duration of in-clinic sessions relative to sensor lifetime.
    Sampling schedules for comparator measurements.
    Glucose and insulin manipulation protocols to test device performance under extreme glycemic conditions.
  Ensure significant glycemic variability is represented.
F. **Data Analysis and Statistical Performance Evaluation**
  Characteristics of comparator data:
    Glucose concentrations, rate of change (RoC), and time course of comparator glucose concentrations
  Protocols for data pairing between CGM and comparator measurements.
  Methods for evaluating key performance metrics:
    Point accuracy (e.g., MARD, consensus error grid).
    Trend accuracy.
    Threshold and predictive alert reliability.
    Technical reliability.
    Sensor-to-sensor variability
    Biases
  Exclusion criteria for data points (e.g., due to failure or protocol deviations).
G. **Reporting and Transparency**
  Publish results in indexed, peer-reviewed journals to ensure scientific rigor.
  Explicitly describe the study methodology, including the setting, clinical context, and comparator methods.
  Include detailed reporting of adverse events and serious adverse events.
  Clearly define and report the number of paired reference readings across anatomical insertion sites
    CGM studies for type 1 diabetes should have > 10,000 paired readings for ages ≥ 18 years old and > 2,500 for ages ≤ 18 years old for each anatomical insertion site [14].
  Provide data on days of sensor use life assessed at the beginning, middle, and end of sensor use to assess performance across its lifespan.
  Report user satisfaction metrics (if applicable).

### Rationale

A 2023 narrative review by Pemberton et al. critically examines the accuracy of CGMs and contrasts the regulatory processes of CE marking in the UK with the FDA (USA) and Therapeutic Goods Administration (TGA) (Australia) [14]. The review finds that FDA and TGA-approved devices undergo more rigorous assessments, leading to better-aligned clinical claims and real-world performance Table 1 [14].

**Table 1:** Regulatory Requirements for CGM Approval and Surveillance: CE Marking (EU & UK) vs. FDA (USA) [14]

Regulatory body	EU and UK (CE marking)	USA (FDA—CDRH)
Approval process	Manufacturer selects a Notified Body accredited by the European Commission and applies for CE marking with a submission dossier containing clinical data	Manufacturer applies to the FDA for Class III (high risk) or iCGM Class II (moderate/high risk) approval
Classification	Class IIa (moderate risk) or IIb (moderate/high risk)	CGM: Class III (high risk); iCGM: Class II (moderate/high risk)
Key requirements	Notified Body assesses conformity based on EU Directive 93/42/EEC (before May 2021) or EU MDR 2017/745 (after May 2021). Evaluation may rely on clinical data from an equivalent device if equivalence is justified. Greater oversight of Notified Bodies is mandated by EU MDR 2017/745	Class III CGM requires a full evaluation of product performance and safety. iCGM (Class II) requires full evaluation against CGM-specific study design and accuracy criteria (QDK, QBJ, QLG)
Approval validity & transparency	CE marking is valid for up to five years. No publicly available document details the submitted evidence or specific indications for use. CE marking certificate is available upon request but lacks indication details	FDA Pre-Market Approval (PMA) is granted for a specific indication (diabetes type and age group) and varies in duration based on classification and code. Approval documents detailing evidence and indications are available in the FDA Medical Devices Database
Post-market surveillance	Manufacturer notifies the MHRA of CE marking and post-market surveillance plans. No pre-market assessment is required by MHRA	Manufacturer’s market and distribute the device in the US and provide post-market surveillance data to the CDRH
User & healthcare provider reporting	Manufacturers trade in the UK and provide post-market surveillance data to MHRA. Users and healthcare providers report issues via the MHRA yellow card system. Updates to CGM technology and indications are not published publicly	Users and healthcare providers report performance and safety issues through the Medical Device Reporting system. Updates to CGM technology and indications for use are published in the FDA Medical Devices Database

Pemberton et al. underscores the need for greater scrutiny of CGM accuracy to enhance patient safety and improve clinical decision-making, and presents a structured approach to evaluating the design, point accuracy reporting, and point accuracy performance of CGM systems, emphasizing the need for methodological rigor in CGM accuracy studies [14]. Pemberton et al., highlights the importance of publication in peer-reviewed journals and the selection of a well-defined population to assess the accuracy of CGMs. The need for paired glucose measurements over a wide glycemic range and at different points in the sensor lifecycle is emphasized. In addition, key metrics such as mean relative absolute difference (MARD), sensor stability, and performance at different glucose levels are analyzed, aligning with FDA and TGA standards. The disparity between regulatory frameworks and the need for stricter criteria in CE marking is highlighted, promoting greater supervision, transparency in reporting and standardization of criteria to ensure safe and accurate devices [14].

Furthermore, a 2023 scoping review by Freckmann et al. on the clinical performance evaluation of CGM systems underscores the urgent need for standardization in study designs and reporting methodologies [3]. While CGM systems play a vital role in diabetes management, inconsistencies in evaluation methods hinder the comparability and reliability of research findings. The review analyzed studies published between 2002 and 2022, identifying significant variability in subject populations, reference measurement techniques, testing protocols, and statistical analyses. Key issues included a lack of uniformity in how comparator glucose readings were collected, low sampling frequency in many studies, and an overreliance on MARD as the primary accuracy metric [3, 5]. The findings highlight that without a standardized criterion, it remains difficult to draw meaningful comparisons between different CGM devices, ultimately affecting clinical decision-making and regulatory oversight.

This review provides a compelling argument for establishing clear guidelines to enhance the consistency of CGM performance evaluations. The recommendations, aligned with the IFCC, emphasize precise design definitions, transparency in the selection of comparators, and statistical rigor. In addition, they highlight the need for collaboration between regulators, manufacturers, and researchers to standardize CGM evaluation and improve diabetes care.

Similarly, in 2024, Mathieu et al. reported the minimum expectations for market authorization of CGM devices in Europe—the “eCGM” compliance status, the study emphasizes the need to establish robust sample size criteria for evaluating the performance of CGM sensors. It suggests that approximately 100 subjects should be included in performance assessment studies to generate a sufficient number of paired glucose readings for statistically significant validation. A typical study design involves three in-clinic visits of 10 h each, with blood samples drawn every 15 min, resulting in approximately 12,000 paired data points for comparison [5, 12]. While the Conformité Européenne (CE) mark is required for medical devices before being marketed in Europe, it does not currently define CGM-specific performance standards. The IFCC is working toward developing ISO standards for CGM accuracy; however, immediate action is needed to users’ safety. The proposed solution is an “eCGM” compliance status, which would require CGM devices to meet stricter, transparent criteria for accuracy, clinical validation, and data integrity, ensuring a basic level of safety across the European market [12].

CGM performance should be proven across the dynamic glycemic range from hypoglycemia to hyperglycemia., stability throughout their lifespan, and validation under real-world conditions (exercise, meals, medication). In addition, they must comply with cybersecurity and data transparency regulations, ensuring secure transmission and detailed information on labeling for informed decision-making [12].

#### Calibration protocols

**Table:** 

Key Points • Factory-calibrated CGMs improve accuracy, reduce user burden, and minimize calibration errors- “factory-calibrated” denotes sensors that are pre-calibrated at the point of manufacture and therefore do not require user calibration to meet their specified accuracy. Some of these systems nevertheless permit optional manual calibration- • Manual calibration introduces variability and requires precise user execution • Clear calibration instructions include recommended glucose meters, optimal timing, and error correction methods • Environmental factors (e.g., hematocrit variations, dehydration) impact calibration accuracy and must be considered • Educational resources and ongoing support help users maintain accurate calibration and improve CGM reliability	

### Recommendations

4. Regulatory standards should prioritize the approval of factory-calibrated CGM systems, as they simplify device use and improve accuracy.
5. Performance studies should explicitly describe the calibration method used for devices requiring manual calibration, ensuring transparency and reproducibility. Manufacturers must provide clear, precise instructions for healthcare professionals and patients on how to perform manual calibration, ensuring safe and effective use.

### Rationale

Factory-calibrated CGM systems significantly reduce the burden on users while improving accuracy, reliability, and adherence to continuous glucose monitoring [18, 23, 29]. Prior CGM systems that require manual calibration introduce potential sources of error, as users must correctly perform capillary blood glucose testing and enter the values accurately [18]. Any deviation in this process—such as improper fingerstick technique, BGM meter inaccuracy [30], or incorrect data entry—can lead to calibration errors that affect glucose readings throughout the sensor’s lifespan. Factory calibration eliminates these challenges by ensuring that each sensor is pre-calibrated during manufacturing, reducing the risk of user-induced variability [12, 17]. As a result, factory-calibrated CGMs provide more consistent readings without the need for frequent manual adjustments, which is particularly beneficial for patients with limited technical skills, cognitive impairments, or those new to diabetes technology. By streamlining the user experience, factory calibration not only enhances device accuracy but also fosters long-term patient adherence, leading to better glycemic control and reduced diabetes-related complications [31, 32].

Beyond accuracy, clear and transparent calibration instructions are essential for ensuring safe and effective CGM use, particularly for systems that still require manual calibration [21, 23, 29]. While factory-calibrated devices are preferred, some CGM models require periodic user calibration, making it crucial that manufacturers provide detailed, step-by-step guidance. Instructions should specify the recommended glucose meters for calibration, optimal timing for blood glucose testing, and how to recognize and respond to calibration errors. Additionally, information about environmental and physiological factors—such as hematocrit variations, dehydration, potential interferences—that could impact calibration accuracy should be explicitly included [33]. Without accurate guidance, users may unknowingly introduce errors that compromise the accuracy of their CGM readings, leading to inappropriate insulin dosing decisions and increased risk of hypoglycemia or hyperglycemia [34, 35].

Educational resources play a pivotal role in promoting correct calibration practices and ensuring CGM effectiveness across diverse user populations [5, 8, 10]. Patients with diabetes vary widely in their health literacy, technological dexterity, and access to diabetes education, meaning that manufacturers must develop materials that are affordable, culturally appropriate, and easy to understand. These resources should include visual aids, instructional videos, and interactive digital content to accommodate different learning styles. Additionally, healthcare professionals must be equipped with training materials that enable them to educate patients effectively on calibration procedures and troubleshooting steps. Providing ongoing support—such as helplines, mobile app guidance, and automated reminders—can further reinforce correct calibration practices, minimizing errors and enhancing user confidence. Ultimately, a well-informed user base ensures that CGM systems function as intended, reducing the likelihood of inaccurate readings and improving overall diabetes management outcomes [4].

#### Report generation and data sharing

**Table:** 

Key Points • ATTD consensus clarified CGM use, endorsing Time in Range (TIR) over HbA1c • Personalized SMART goals improve self-management and reduce hypoglycemia • Access barriers to CGM can be reduced through simple, standalone reader devices • Clear communication of software updates is essential for safe and effective use • Educational resources should support proper calibration practices to enhance device reliability	

### Recommendations

6. CGM reports should be integrated into a digital ecosystem that facilitates seamless communication and data sharing among healthcare professionals, users, and caregivers/family members.
7. Reporting standards should adhere to the minimum recommendations by the 2019 international consensus of the Advanced Technologies & Treatments for Diabetes (ATTD) [17].
8. Manufacturers should standardize language and unit customization in CGM reports.
9. A robust mechanism to protect sensitive user data and compliance with local data privacy regulations should be provided.

### Rationale

Formal uptake into professional guidelines has been limited until recently; Chapter 8 of the 2022 ISPAD Clinical Practice Consensus Guidelines, aimed at children, adolescents and young adults—now endorses specific CGM targets (e.g., ≥ 70% time in range 70–180 mg/dL, ≤ 4% < 70 mg/dL) [36].

A key outcome of the consensus panel was the endorsement of “time in ranges” (TIR) as a superior metric for glycemic control compared to HbA1c alone [17]. TIR, is the percentage of time that glucose levels remain within a specified target range, typically between 70 and 180 mg/dL for individuals with diabetes [37]. The panel provided specific TIR targets tailored to different diabetes populations, including type 1, type 2, pregnant women, and older individuals at higher risk. Additionally, the panel recommended using the Ambulatory Glucose Profile (AGP) as the standardized visual report for interpreting CGM data [17]. Standardized measures help clinicians and patients discuss glucose trends effectively, enabling timely, informed diabetes management adjustments. The adoption of these recommendations is expected to enhance the clinical utility of CGM data, translating technological progress into tangible improvements in diabetes care [37].

A fundamental principle emphasized by the panel is that goal-setting must be individualized, taking into account the unique needs and capabilities of each person with diabetes. Studies have demonstrated that setting small, realistic goals not only improves diabetes self-management but also fosters long-term behavioral changes [17]. The use of SMART (Specific, Measurable, Achievable, Relevant, Time-bound) framework was identified as a useful tool for establishing CGM-based targets, reducing the time in hypoglycemia to less than one hour daily, facilitating adherence [17]. This highlights the need for personalized, stepwise interventions and a collaborative approach in diabetes management.

For specific populations, such as children, adolescents, and young adults, the panel recognized that glycemic targets must balance optimal glucose control with considerations of safety, quality of life, and burden of care. For women planning pregnancy or those already pregnant, early attainment of glycemic targets is crucial for both fetal and maternal health [17].

On the other hand, ensuring accessibility for all users is a fundamental aspect of equitable diabetes management, particularly when considering CGM technology. While smartphone-based CGM systems provide convenience and enhanced data analysis capabilities, their implementation requires careful consideration [8, 12]. This digital device disproportionately affects older adults, individuals from lower socioeconomic backgrounds, and those living in regions with limited access to advanced technology. To bridge this gap, manufacturers should provide standalone reader devices as an alternative to smartphone integration [25, 27]. These dedicated readers should be designed with simplicity in mind, featuring clear displays, large fonts, and intuitive navigation to accommodate individuals with visual impairments or limited technological literacy. Additionally, reader devices should be made widely available through healthcare systems, insurance providers, or patient assistance programs to ensure affordability and prevent financial barriers from limiting access to CGM technology [38]. By offering standalone readers alongside smartphone compatibility, manufacturers can uphold access in diabetes care, ensuring that all individuals, regardless of technological access, can benefit from continuous glucose monitoring [39].

The definition and design of CGM reader devices play a critical role in enhancing usability and accessibility for diverse patient populations. A CGM reader must, at a minimum, display real-time glucose readings, trend arrows, and optional alerts for hypo- and hyperglycemia to support immediate decision-making [3, 6, 12, 25]. However, to maximize effectiveness, readers should also incorporate additional features such as customizable alerts, insulin dose tracking, and trend analysis to provide users with a more comprehensive understanding of their glucose patterns. Additionally, manufacturers should consider alternative formats for CGM readers, such as voice-assisted devices for visually impaired users or wearable technology that allows discreet glucose monitoring [40].

Transparent and effective communication of software updates is crucial to maintaining the safety and usability of CGM systems. Software updates frequently include security enhancements, bug fixes, algorithm improvements, and new features that can significantly impact device performance [18, 27]. However, if these updates are not properly communicated, users and healthcare professionals may be unaware of critical changes, potentially leading to confusion, errors in device operation, or unintended interruptions in glucose monitoring [1]. Furthermore, any major software changes should be accompanied by user-friendly guidance explaining how to navigate new features or resolve potential issues. Healthcare professionals should also receive advance notice and educational resources to ensure they can provide adequate support to their patients. By prioritizing transparency in software updates, manufacturers can foster user trust, prevent misinformation, and ensure that CGM systems continue to function optimally in real-world settings.

#### User support, post-sale services, and patient safety

**Table:** 

Key Points • Reliable technical support ensures CGM functionality and user trust • Full disclosure of materials is needed to identify allergens and interactions • Customizable alarms and signal loss alerts improve patient safety • Transparent reporting enhances regulatory compliance and user confidence • Compliance with international biocompatibility standards (e.g., ISO 10993) is essential to ensure material safety and minimize adverse reactions • Strong data security and regulatory compliance—CGM manufacturers must implement robust encryption and privacy safeguards to prevent unauthorized access or data breaches, ensuring compliance with GDPR and HIPAA to protect user confidentiality • Structured audits and post-market surveillance—Periodic performance audits and integration of real-world data are essential to maintaining CGM accuracy, mitigating risks, and reinforcing public trust in device safety and reliability	

### Recommendations

10. Manufacturers should provide accessible technical support and customer service channels in each country where the device is marketed, ensuring a continuous omnichannel system. A robust post-sale warranty system should be established, including clear device replacement.
11. The device should comply with ISO 10993 international standards that assess the biocompatibility of medical devices and explicitly report materials, potential allergens, and possible interactions with medications or other devices, such as imaging systems, airport scanners, or implantable devices.
12. Devices must include customized alarm thresholds based on individual needs and alerts for signal loss to inform users promptly and mitigate risks.

### Rationale

Comprehensive user support is essential to ensuring the successful adoption and sustained use of CGM systems, particularly given their critical role in diabetes management [38]. A robust omnichannel customer service framework allows users to access timely solutions across multiple platforms, including phone support, online chat, email, and dedicated mobile applications. This level of accessibility is crucial, as CGM users may encounter technical issues that require immediate resolution to avoid disruptions in glucose monitoring [41]. Moreover, language accessibility and culturally competent support services must be prioritized to ensure that users from diverse backgrounds can effectively communicate their concerns and receive appropriate guidance. Beyond technical troubleshooting, customer support should extend to education and training, empowering users to maximize the benefits of CGM technology [26]. When manufacturers provide reliable, responsive, and accessible customer service, they foster trust in their devices, encourage long-term adherence, and ultimately contribute to improved diabetes outcomes.

Transparency in product information is a fundamental principle that directly impacts user safety and decision-making. Users and healthcare professionals must have access to comprehensive details regarding CGM device materials, potential allergens, and interactions with medications, implantable medical devices, or external factors such as MRI scans or airport security scanners [5]. Failure to disclose this information could lead to adverse reactions or compromised device functionality, putting users at risk. Additionally, regulatory agencies and independent researchers must be able to scrutinize these disclosures to ensure compliance with safety standards [5]. By adopting a transparent approach, manufacturers can strengthen consumer confidence, promote informed decision-making, and align with ethical standards that prioritize patient safety. Clear, publicly available product information also enhances the credibility of CGM technology within the medical community, encouraging wider adoption and integration into clinical practice.

Quality assurance is a cornerstone of CGM device reliability, and periodic audits play a vital role in maintaining high performance and safety standards. These audits are essential components of quality management systems, ensuring that manufacturers comply with international standards [3, 42]. Furthermore, post-market surveillance data should be incorporated into these evaluations, allowing real-world user feedback to inform necessary design improvements. By enforcing a structured audit process, manufacturers can demonstrate accountability, mitigate risks associated with device malfunction, and reinforce public trust in CGM technology as a dependable tool for diabetes management.

Safety features, particularly signal loss alarms when available, are indispensable in preventing glucose data gaps and ensuring continuous data availability [1]. CGM users rely on real-time glucose readings to make critical treatment decisions, and any unexpected loss of sensor connectivity can pose serious health risks, especially for individuals with insulin-dependent diabetes. Signal loss alarms serve as an immediate alert mechanism, notifying users of connectivity issues so they can take corrective action before gaps in monitoring lead to hypo- or hyperglycemia [20, 38]. Additionally, manufacturers should provide clear guidance on troubleshooting steps and, where possible, incorporate redundancy measures such as automatic reconnection protocols.

Bioethical concerns surrounding CGM technology must be carefully considered, particularly regarding data privacy, equitable access, and informed consent [43]. As CGM systems collect and transmit sensitive health data, manufacturers must implement robust security measures to prevent unauthorized access or data breaches. Compliance with local and international privacy regulations, such as GDPR or HIPAA, is crucial to protecting user confidentiality [12, 43]. Beyond data security, equitable access remains a pressing issue, as CGM devices are often cost-prohibitive for many patients, exacerbating health disparities [44]. Manufacturers should work towards making CGM systems more affordable through cost-reduction strategies, insurance coverage expansion, and assistance programs. Additionally, informed consent should be a continuous process, ensuring that users fully understand how their data is collected, shared, and used for product improvement or research [12, 17, 43]. By addressing these bioethical concerns proactively, CGM manufacturers can uphold ethical standards while ensuring that their technology benefits the widest possible patient population.

### Checklist

To ensure practical implementation, the expert panel developed a regulatory checklist (Table 1) summarizing essential criteria CGM devices should meet. This checklist outlines key performance metrics, study design elements, calibration specifications, reporting standards, and safety measures.

## Discussion

The regulatory landscape for CGM devices in Latin America has challenges to the standardization of device accuracy, reliability, and clinical effectiveness. This consensus statement asserts the urgent need for a standardized regulatory framework in the region to address device accuracy, reliability, and patient safety inconsistencies. The modified Delphi methodology adopted in this study sought to address these gaps by engaging regional experts to develop a consensus-driven regulatory framework. By leveraging real-world expertise, this approach ensures that the proposed standards align with both clinical practice and patient needs while considering the unique challenges of the region’s healthcare landscape.

The expert panel proposes 12 key recommendations to bridge the gap between Latin American and international CGM device standards, including establishing minimum accuracy thresholds, standardizing performance study designs, and ensuring transparent reporting of real-world device performance. These recommendations align closely with FDA iCGM performance requirements [5] and the minimum expectations for market authorization of CGM devices in Europe [12], emphasizing stringent accuracy benchmarks and comprehensive clinical validation protocols.

We highlight the importance of rigorous pre-market and post-market evaluations, requiring manufacturers to adhere to accuracy thresholds that reflect real-world usage. Moreover, the recommendations stress the necessity of continued oversight and quality audits to verify long-term device performance [12, 17]. By establishing a structured regulatory requirement, policymakers can enhance the consistency and safety of CGM technology, ultimately fostering a more robust and patient-centered approach to diabetes management.

Beyond regulatory improvements, we also underscore the importance of patient accessibility and education in maximizing the benefits of CGM technology [38, 43]. Factors such as affordability, ease of use, and integration with digital health platforms significantly impact CGM adoption rates and patient adherence [44]. The expert panel emphasized the need for clear, standardized patient education materials and training programs to ensure users fully understand how to interpret and respond to CGM data. Additionally, manufacturers must prioritize the development of user-friendly interfaces and data-sharing capabilities that facilitate seamless communication between patients and healthcare providers [8, 17]. Addressing these challenges holistically—through regulatory standardization, methodological rigor, and enhanced patient support—will ensure that CGM technology reaches its full potential in Latin America.

This consensus statement is strengthened by the use of a structured expert consensus methodology, ensuring that recommendations are informed by the perspectives of leading endocrinologists, pediatric endocrinologists and diabetologists across Latin America. The modified Delphi approach enhances the validity of the proposed regulatory framework by incorporating real-world clinical expertise and addressing regional healthcare challenges. Furthermore, the recommendations align with international best practices, facilitating potential harmonization with established regulatory frameworks such as the FDA iCGM performance requirements [5, 12]. However, limitations exist, including the relatively small number of participating experts, which may not fully capture all perspectives within the Latin American healthcare ecosystem. Additionally, while the proposed framework is designed for broad applicability, implementation feasibility may vary across countries due to differences in healthcare infrastructure, regulatory capacity, and economic constraints. Furthermore, a recent letter from the IFCC Working Group on CGM [45] warns that directly transplanting FDA iCGM special-control requirements into other jurisdictions can create unforeseen barriers, emphasizing the importance of local adaptation.

Future research should focus on defining actionable steps for regulatory authorities in Latin America to establish standardized accuracy and performance requirements for CGM systems.

## Conclusion

The absence of standardized regulatory guidelines for CGM devices in Latin America presents a barrier to ensuring device accuracy, reliability, and clinical safety. This consensus statement highlights the urgent need for a structured regulatory framework aligned with international best practices, advocating for minimum accuracy thresholds, standardized clinical validation protocols, and robust post-market surveillance. Additionally, improving patient education and accessibility remains a crucial component of successful CGM implementation. By addressing these multifaceted challenges, Latin America can establish a more effective and equitable approach to CGM regulation, ultimately improving diabetes management and patient outcomes throughout the region.

## Data Availability

No datasets were generated or analysed during the current study.

## Appendix

See Table 2

**Table 2:** Checklist—key performance metrics, study design elements, calibration specifications, reporting standards, and safety measures for CMG devices in Latin America

Criteria		Yes	No	Comment
1. Accuracy criteria				
1	Precision and accuracy are reported using the following metrics: Mean Absolute Relative Difference (MARD), consensus error grid, and percent of results within a defined range vs reference			
1.1	**MARD**: The device demonstrates a Mean Absolute Relative Difference (MARD) < 10% when compared with venous blood glucose			
1.1.1	MARD is reported throughout the sensor’s operational lifespan, considering different glucose thresholds such as hypoglycemia, hyperglycemia, and Time in Range (TIR)			
1.2	**Consensus error grid:** Report results in Zones A and B, in which have no effect on the clinical action or the action would have little effect on the clinical outcome			
1.3	**Agreement rate**: Agreement thresholds for iCGM accuracy: Each item below specifies the minimum percentage of acceptable agreement relative to blood glucose values across defined glycemic ranges [5, 14]: **iCGM special control performance requirement (all with 95% LCL*):** • A: Within 15 mg/dL, < 70 mg/dL: > 85% • B: Within 15%, 70–180 mg/dL: > 70% • C: Within 15%, > 180 mg/dL: > 80% • D: Within 40 mg/dL, < 70 mg/dL: > 98% • E: Within 40%, 70–180 mg/dL: > 99% • F: Within 40%, > 180 mg/dL: > 99% • G: Within 20%: > 87% • H: CGM < 70 mg/dL & Ref** > 180 mg/dL: 0 • I: CGM > 180 mg/dL & Ref < 70 mg/dL: 0 • J: CGM ROC*** > 1 mg/dL/min & Ref ROC < –2 mg/dL/min: < 1% • K: CGM ROC < –1 mg/dL/min & Ref ROC > 2 mg/dL/min: < 1% *LCL: lower confidence limit, **Ref: reference glucose value, ***ROC: rate of change (glucose rate of change in mg/dL per minute)			
2. Design of performance studies				
2.1	**Population Representation:** Performance is validated in diverse populations, including pediatric, adult, older adults, and pregnant individuals*			
2.2	**Anatomical Sites:** Performance is reported for all intended anatomical application sites, including paired readings			
2.3	**Study Setting**: Performance studies clearly describe whether the study was conducted in-clinic, free-living, or hybrid setting			
2.4	**Comparator measurement**: Sampling compartment (capillary, venous, or arterialized-venous) is clearly defined			
2.5	**Reporting**: Performance studies are published in indexed, peer-reviewed journals			
2.6	**RWE**: If approved, performance is reported for everyday use, including high-stress environments (e.g., hospitals)			
3. Calibration				
3.1	**Factory-calibrated**: The device is factory-calibrated and calibration remains accurate throughout the sensor’s lifespan			
3.2	**Calibration method:** If the device requires manual calibration, the calibration method is explicitly described and precise instructions for healthcare professionals and patients on how to perform manual calibration is provided			
4. Report generation and data sharing				
4.1	**Standardized reports**: The report adheres to the recommendations by the 2019 international ATTD consensus [14, 17]			
4.2	**Interoperability**: The device integrates seamlessly with smartphones, insulin pumps, and other diabetes management devices			
4.3	**Privacy compliance**: The device adheres to local data privacy regulations			
5. User support, post-sale services, and patient safety				
5.1	**Technical support**: Manufacturer provides omnichannel customer support in every market where the device is available			
5.2	**Warranty and repairs**: Manufacturer ensure robust post-sale policies, including user-friendly educational resources, warranties, and device replacement protocols			
5.3	**Biocompatibility**: The device complies with standards and explicitly reports materials, potential allergens, and interactions with medications or other devices			
5.4	**Customizable alerts**: The device allows users to customize alarm thresholds and settings based on individual needs			
5.4.1	**Alerts metrics**: True alert, false alert, missed alert, and correct alert rates are provided			
5.5	**Signal loss:** The device includes alerts for signal loss			

*The population representation is for the intended approval

## Abbreviations

ATTD: Advanced technologies & treatments for diabetes
BGM: Blood glucose monitoring
BMI: Body mass index
CDRH: Center for devices and radiological health
CGM: Continuous glucose monitoring
FDA: Food and drug administration
GDPR: General data protection regulation
HbA1c: Hemoglobin A1c
HIPAA: Health insurance portability and accountability act
iCGM: Integrated continuous glucose monitoring
IFCC: International federation of clinical chemistry and laboratory medicine
ISO 10993: International organization for standardization 10993
MARD: Mean absolute relative difference
ROC: Rate of change
TIR: Time in range
