# Correction: Consensus statement on standardizing CGM evaluation metrics in Latin america: an expert approach

**DOI:** 10.1186/s13098-025-01997-x

**Published:** 2025-11-20

**Authors:** Mauro Scharf, Karen Feriz, Carlos Yepes, Álvaro Contreras, Lorena Lequi, David Sanhueza, Marcio Krakauer, Eduardo Márquez, Matías Ré, Roopa Mehta

**Affiliations:** 1 Pediatric Endocrinology Unit, Centro de Diabetes Curitiba, Hospital Nossa Senhora das Graças, Curitiba, Brazil; 2 Endocrinology Service, Fundación Valle del Lili University Hospital, Universidad ICESI, Cali, Colombia; 3 Department of Endocrinology, Hospital Universitario Clínica San Rafael and Hospital Universitario San Ignacio, Bogotá, Colombia; 4 Department of Diabetology, Clínica Universidad de los Andes, Santiago, Chile; 5 Mains Bleues Diabetes, Centro de Investigación diabetes, metabolismo y enfermedades asociadas, Rafaela, Santafe Argentina; 6 Department of Diabetology, Santa Fe, Argentina; 7 Department of Diabetology, Universidad Andrés Bello and Hospital y CRS El Pino, Santiago, Chile; 8 Diabetes Division, Liga de Diabetes, Faculty of Medicine, ABC Medical School, São Paulo, Brazil; 9 Department of Endocrinology, Instituto Jalisciense de Metabolismo, Guadalajara, Mexico; 10 Department of Diabetology, CINME (Centro de Investigaciones Metabólicas), Buenos Aires, Argentina; 11 Department of Endocrinology and Metabolism and UIEM, Instituto Nacional de Ciencias Médicas y Nutrición Salvador Zubirán, Mexico City, Mexico

**Correction: Diabetology & Metabolic Syndrome (2025) 17, 291**

10.1186/s13098-025-01851-0

In the original publication of this article [1], an incorrect description of the IFCC Working Group on CGM recommendations was included in Results sections. The incorrect and correct description are displayed below: 

In Results section, under fifth paragraph of “Rationale” heading, the sentence should read, “In line with the IFCC Working Group on CGM [16], at least 7.5% of all values should cover each of the following situations:

- BG< 70 mg/dL;
- BG>300 mg/dL;
- BG≥ 70 mg/dL with rate of change < –1 mg/dL/min or BG < 70 mg/dL within 30 minutes at the current rate of change; and
-  BG ≤ 300 mg/dL with rate of change >+1.5 mg/dL/min or BG >250 mg/dL within 30 minutes at the current rate of change” instead of “In line with the IFCC Working Group on CGM, at least 20% of paired samples should be obtained within each predefined rate-of-change stratum (≤ 1, 1–2, and >2 mg dL⁻¹ min⁻¹) [16]”.

The original article has been corrected.
